# Stem cells part of the innate and adaptive immune systems as a therapeutic for Covid-19

**DOI:** 10.1080/19420889.2021.1965356

**Published:** 2021-09-10

**Authors:** Greg Maguire

**Affiliations:** Dept. of Preventative and Medicinal Chemistry, NeoGenesis Inc. And BioRegenerative Sciences Inc, San Diego, CA, USA

**Keywords:** Adult stem cells, immune system, adipose derived mesenchymal stem cells, Covid-19, inflammation, viral infection

## Abstract

Some stem cell types not only release molecules that reduce viral replication, but also reduce the hypercytokinemia and inflammation induced by the immune system, and have been found to be part of the innate and adaptive immune systems. An important component of the stem cell’s ability to ameliorate viral diseases, especially the complications post-clearance of the pathogen, is the ability of adult stem cells to reset the innate and adaptive immune systems from an inflammatory state to a repair state. Thus, the molecules released from certain stem cell types found to be safe and efficacious, may be an important new means for therapeutic development in Covid-19, especially for late-stage inflammation and tissue damage once the virus has cleared, particularly in the aged population.

## Introduction

Elimination of the pathogen or creating herd immunity are the best means to protect the population from viral infections such as SARS-CoV-2, a virus with very sophisticated gene expression and replication mechanisms, capable of extensive remodeling of its intracellular environment, and possessing multi-faceted immune evasion strategies. However, in diseases such as Covid-19 where the adaptive immune system of older adults is less robust than that of younger people, and where the population is increasingly aging, the goal of herd immunity may be difficult to meet as the older people may not respond well to Covid-19 vaccines; not true for the new shingles vaccine however (1). The durability of a vaccine for Covid-19 is not yet known, and secondary humoral and cellular immune responses have not yet been thoroughly studied (2). Several recent studies have found that Covid-19 patients can be reinfected, raising further concern that immunity against SARS-CoV-2, whether natural of through vaccines, can be durable (3; 4). Difficulty may also be the result of the anti-vaxxer movement where a recent U. Chicago poll found that 20% of the respondents say they will not be vaccinated and another 31% are not sure (5). Further, even if herd immunity is established through vaccination, the intermediate vector between bats, pangolins and humans leading to spillover to humans has not been identified (6), and along with significant mutation rates, possibly host induced (6), may mean that vaccinations for SARS-CoV-2 will only be good for a limited population and period of time; thus, older susceptible individuals may remain at risk for Covid-19. COVID-19 patients with moderate disease displayed a progressive reduction in type-1 (antiviral) and type-3 (antifungal) responses. In contrast, patients with severe disease maintained these elevated responses throughout the course of disease (7). Thus, a phased treatment plan may be important for Covid-19, where early phase treatment may include interferon and antivirals to kill the virus and later phase treatment includes resolving inflammation (8) and repairing tissue damage such as fibrosis. Recent studies suggest, for example, that IFN-λ may be effective early in the disease to reduce viral replication, but acts on lung epithelial cells late in the infection to degrade lung barrier function, predisposing the patient to secondary lung infection (Broggi et al, 9). Moreover, those that have had Covid-19 and survived, often have significant long-term morbidities, many of which may be effectively ameliorated with the proposed therapeutic. In a related coronavirus disease, SARS in 2003, in patients who were found to have lung lesions 1 year after infection still had lesions after 15 years [10]. SARS-CoV-2 is causing lingering (months) symptoms for many patients, and studies are now underway to follow patients for at least two years in order to understand long lasting, possibly debilitating, symptoms (11). While many companies and physicians continue to use stem cells and the molecules/exosomes derived from stem cells, the vast majority of these therapeutics are unproven and often dangerous (12). Further, bringing patients into a clinical setting, especially for infectious diseases, can lead to superspreading of pathogens even in the world’s most prestigious hospitals such as the National Institute of Health hospitals in the USA (13). The problem confronts us in the current pandemic where physicians have reported that hospitals are the ‘main Covid-19 carriers’ of the SARS-CoV-2 virus (14). In a modern hospital at the Univ Florida where Covid-19 patients are being treated, aerosolized SARS-CoV-2 virions, capable of being cultured, were found throughout the patient rooms, at least 16 ft from the patient (15). Thus, non-clinical administration of a therapeutic would obviate one important means of SARS-CoV-2 transmission.

Even in those therapeutics that are FDA approved, pharma-funded studies are highly biased and yield more positive results than do public-funded clinical trials (16). Consequences of industry bias in the approval process include post-market approval withdraw of therapeutics because of significant adverse events (17) and drugs that lack efficacy, where, for example, in the USA,18 of 36 FDA approved cancer drugs using surrogate endpoints were shown to have no effect on the cancer (18). To help change the landscape of a corrupt healthcare system (19), stem cell-based therapeutics that are safe and efficacious as determined by rigorous preclinical and clinical studies leading to an FDA approval is needed. Because companies (20) and physicians (21; 22) leading these therapeutic development programs are often biased or cheat because of financial incentives, about half of the drugs on the market don’t work (23) and half of medical procedures don’t work (24; 25). Better public sector oversight is needed to assure safe and efficacious stem cell therapeutics are brought to market.

## Stem Cells as part of the Immune System

Stem cells have been found to be part of the innate and adaptive immune systems. Some stem cell types not only release molecules, most of which are packaged into exosomes, that reduce viral replication, but also reduce the hypercytokinemia and inflammation induced by the immune system. Thus, stem cell therapy can be thought of more as medical chemistry than as cellular therapy because the molecules released from stem cells provide most of the therapeutic benefit in fighting viral infections and the resulting cytokine storm as exemplified in Covid-19. While public health measures are our first line of and most important means of defense in the newly emerging Covid-19 pandemic, and comorbidities and exposome of the individual may be important factors in susceptibility to infection and its severity (25), the development of new antimicrobials, along with vaccines, are additional means of potentially fighting this infectious agent, coronavirus SARS-CoV-2. Stem cells and the molecules that stem cells release may be an important new means of developing such antimicrobial therapeutics because the molecules that stem cells release can act in two ways, 1. to inhibit viral replication or entry into host cells, and 2. Resolve the inflammatory damage to host cells that was induced by the virus, the so-called “cytokine storm.” And, being a natural component of the body, these molecules, if chosen from the proper stem cell types (26), may have few if any negative side effects [27]. Although premature, stem cell treatments in clinical trials for Covid-19 infections are currently underway in China and the USA, and other locations. . (28) reported the safe and efficacious use of ADSCs for treatment of patients with pneumonia and being ventilated, and a review of the clinical trials testing mesenchymal stem cell-based therapeutics for Covid-19 has been presented by 29. The safety concerns for stem cell-based therapeutics and their mitigation, especially as related to Covid-19, has been presented by 30.

Let’s consider the rationale for using stem cells to fight a viral infection such as Covid-19. But first, consider why we may not want to use stem cells, particularly some types of stem cells. BMSC transplants may cause the tissue in the host to age; specifically, the very cells needed to fight the infection, the T-cells, have been found to express p16 markers for advanced aging following bone marrow stem cell transplants (31] [32]. Therefore the adaptive immune system that is needed to fight the Coronavirus infection may be compromised by the stem cell transplant. This doesn’t happen when the molecules from adipose derived mesenchymal stem cells (ADSCs) are used instead of the bone marrow stem cells themselves [33], Hong et al.]. Moreover, bone marrow stem cells (BMSCs) have been found to be contaminated with recirculated cancer cells and other phenotypes that may induce untoward cancerous effects [34], as do the molecules that the BMSCs release [35]. Moreover, a number of studies have found that the bone marrow functions as a reservoir for disseminated tumor cells [36]. There is no widely accepted staining protocol that has the necessary specificity and sensitivity to accurately detect cancer cells in a large sample of BM cells [37]. Therefore, BMSC samples may be contaminated with cancer cells. BMSCs have been found to be the cellular origin of certain chromosomal translocation‐associated solid tumors [38], and a number of studies have suggested that the delivery of the FLI‐1/EWS fusion protein into BMSCs may cause transformation of these cells into malignant sarcoma cells [39].

Using the exosomes from BMSCs and those from placenta mesenchymal stem cells (PMSC) may be risky for another reason given that these exosomes express ACE2 and proteases [40]. Because the SARS-CoV-2 virus uses the ACE2 receptor and a protease for entry into the host cell, entry of viral RNA into transplanted exosomes derived from BMSCs and PMSCs is therefore possible. Viruses are well known to highjack the endosomal pathway leading to the production of exosomes, with the ensuing spread of their virion components such as proteins and RNA to other cells [41]. In HIV, the virus has been suggested to have evolved to exploit the exosome system to infect cells in the absence of receptor-mediated interaction. Therefore, if the SARS-CoV-2 viral RNA is contained in an exosome, then that viral RNA could be transmitted by a non-receptor mediated fusion [42] to cells other than those expressing ACE2. If this is the case, Covid-19 could be spread to organ systems not normally infected in the disease. Therefore the risk that the virus will gain direct entry to the BMSC and PMSC exosomes that have already been released from the cell, and are being used for exosome transplants, requires study. Further, BMSCs [43] and PMSCs [44] have been found to be particularly susceptible to viral infections, thus these cells require careful pathogen screening to be sure they are virus-free.

Now for the rationale. Beyond their well described immune modulating effects, where inflammation can be resolved in many organs, and specifically addressing acute respiratory distress syndrome (ARDS), the total collection of molecules released from MSCs can reduce lung inflammation, as well as restore alveolar fluid clearance, and exert antimicrobial activity party through keratinocyte growth factor (KGF) secretion [45,46]. As part of the innate immune system, stem cells release peptides, known as antimicrobial peptides (AMPS) [47] that have been found to fight viral infections [48], including the respiratory infection influenza A [49]. Although for 25 years the prevailing paradigm has been that AMPs are generally nonspecific and functionally redundant, recently AMPs have been found to exhibit high levels of specificity, with functionally important point mutations underlying specificity [50]. Thus, genetic variability in AMPs, even in single amino acids, can dramatically alter resistance to infection. Stem cells are activated by viral infections to release these AMPs, and, interestingly, the stem cells themselves are protected from the viral infection and remain normally active cells even when infection affects the tissue compartment in which the stem cell resides [51]. Exosomes secreted from umbilical mesenchymal stem cells (uMSCs), a cell type widely used in regenerative medicine studies, inhibited Hepatitis C virus infection *in vitro*, especially through inhibition of viral replication, with low cell toxicity compared to other antiviral agents [52]. 53,found that infection with Zika virus dramatically increased IFN-β expression in neural stem cells (NSCs) and that either induction or treatment with IFN-β is able to inhibit Zika virus (ZIKV) replication in NSCs. They also found that the cytoplasmic pathogen sensor RIG-I, a cytosolic pattern recognition receptor (PRR) responsible for the type-1 interferon (IFN1) response, mediates IFN-β transcription in ZIKV-infected NSCs. Because these authors also found that treatment with IFN-β induced downstream antiviral interferon stimulated genes (ISGs) in NSCs, such as ISG56 and MxA, these data suggest that NSCs have intact RIG-I antiviral signaling pathways that are capable of interfering with viral propagation. Whether the NSCs, like other cells [54], including stem cells [52], secrete antiviral molecules and exosomes has not been reported.

Viral infections, including Covid-19, elicit a dramatic increase in serum amyloid A [10], an inflammatory mediator found to be a factor in inducing thrombosis [55]. A case study using autopsy of 5 severe Covid-19 patients found complement associated microvascular injury and thrombosis in the pathogenesis of the infection [56]. The exosomes from ADSCs, more so than those from BMSCs, have been found to decrease both synthesized and secreted β-amyloid peptide [57]. The exosomes from ADSCs were found to contain neprilysin (NEP), an important Aβ-degrading enzyme in the brain [58]. Thus, this may be another important mechanism by which ADSC exosomes resolve inflammation during the “cytokine storm” elicited by the SARS-CoV-2 infection.

Stem cells may play an important role as a part of the adaptive immune system too. Recent studies from Shruti Naik at NYU and Elaine Fuchs at Rockefeller University found that if patches of skin in mice were wounded, causing inflammation, then allowed to heal, subsequent wounds in the same patch of skin would heal about 2.5 times more quickly than adjacent, previously unwounded skin [59]. The effect in previously wounded skin could last up to six months in mice given the conditions of the experiment. Six months of memory in the mouse may be the equivalent of about 15 years for a human. This functional adaptation was attributed to epithelial stem cells (EpSCs) and did not require a canonical immune response because skin-resident macrophages and T cells were not involved. This means that some adult stem cells have immune memory, or at least a memory of past inflammatory events that allows the stem cells to differentially control the immune response. Stem cells with specific memories of immune events may be harnessed in the future to treat specific immune conditions.That is, if a stem cells has been exposed to a wounding or inflammatory event, will that stem cell possess a better phenotype with which to quell the inflammation and heal the wound? What the study found was that EpSCs maintain chromosomal accessibility, where the DNA is less tightly packed and open to signals from the damaged tissue, at key stress response genes, activated by the inflammatory stimulus. This epigenetic change in the chromatin allowed, during a secondary inflammatory challenge to the same skin patch, genes in that patch of skin to be transcribed rapidly. While the secretome of skin stem cells has previously been shown to be altered by wounding, the exact nature of changes in the secretome was not reported in this study. However, underlying the memory of the stem cells in this study is Aim2, a portion of DNA that encodes an activator of the inflammasome, a conglomerate of proteins that contributes to the skin’s defense against bacteria and viruses. More recently, the Fuchs lab revealed that independent of immune cells, stem cells within a damaged niche in the skin release a complex array of cytokines and chemokines to communicate with both the adaptive and innate immune systems [60]. The chemokines include CCL1 [62–64] and CCL20 [65,66,68], which are both potent stimuli for Tregs. Importantly, [60,61],found that stem cells can sense when their niche barrier is breached and produce signals to recruit specific immune cell sentinels, even under conditions where the immune system itself has been suppressed. Similar to stem cells, progenitor cells, fibroblasts, can acquire a trained immunity-like phenotype. [67],found that IFNβ treatment of mouse embryonic fibroblasts led to faster and increased induction of interferon-stimulated genes that correlated with enhanced recruitment of polymerase II to interferon-stimulated gene loci when stimulated again. Although fibroblasts are known to release exosomes with a complex molecular cargo, including proteins and miRNAs, the trained immunity in these cells has not been reported in terms of exosome content.

An emerging area of research is quickly expanding as scientists continue to explore stem cell memory, and the field of immune-stem cell interactions, and stem cells as a part of the immune system. The stem cell functions described here also means that your health is not just genetic. What you do, including wounding yourself or having an infection may have long term consequences to your health, induced not only through mechanisms in the classic immune system, but also induced in immune-like stem cell function. And optimizing your health, including through diet, may help to reset the adaptive immune system to fight viral infection and the resulting inflammation [69]. This study found that fiber added to the diet increased T-cell function to fight viral influenza infection. Adaptive immune cells have memory, and given their adaptation-dependent phenotype, they can release exosomes that reflect their adaptation. For example, T cells have memory for 10 years, and Treg-derived exosomes induce the differentiation of other T cells to the Treg phenotype [70]. Whether stem cells can alter their exosome cargo to a specific set of immune chemicals dependent on adaptation to infection has not been studied as it has with immune cells.

In another study, [51]and colleagues discovered that stem cells are hardwired to express antiviral interferon-stimulated genes (ISGs) that help to fight viral infections. Further, β-glucan, a bacterial and fungal cell wall component induced IL-1β release, which was capable of training both hematopoietic stem cell (HSCs) and myeloid progenitors. These conditioned HSCs and myeloid progenitors were able to more efficiently ward off inflammatory challenges when compared to naïve HSCs. Further, IL-1β-trained HSCs exhibited dramatic changes in their energy metabolism, displaying augmented glycolysis and cholesterol biosynthesis, adjustments that turned out to be critical for conferring downstream functional changes in β-glucan-dependent HSC training [71]. Combine this stem cell training with conditioning of the canonical adaptive immune cells, T-cells, through a high fiber diet that induces a allostatic state [72] and is pro-resolving for viral infections [69], then a synergistic efficacy can occur.

## Inflammation and infection prevention – proresolving molecules

An increase in innate immunity and chronic inflammation or inflammaging is characteristic of aging and of the chronic diseases of aging. Inflammaging results in damage to inflamed tissues and thus decreasing their resilience and response to infection [65,73]. The inflammasome was found to be activated in moderate and severe Covid-10 patients who were autopsied [74]. One key factor determining a poor prognosis, including mortality and long term morbidity, during COVID-19 is inflammation and a hyperactive innate immune system associated with an increase in senescent cells of the respiratory tracts [32], thereby possibly explaining the increased complications in older adults. Increased serum levels of several inflammatory cytokines and chemokines have been associated with death and disease severity, including lung fibrosis [75]. Senescent cells, including in the lungs [76], increase in number as we age and are a factor in the induction of inflammation. Immunostaining of postmortem tissue from patients who had died from COVID-19 found that populations of CD169^+^ lymph node subcapsular and splenic marginal zone macrophages express the SARS-CoV-2 entry receptor ACE2 and that these macrophages contained SARS-CoV-2 nucleoprotein [77. A benefit of ADSCs and their released molecules is a reduction in age related muscosal immunusuppression through an increase of antigen-specific protective SIgA antibody responses (78], important to the early phase of interrupting SARS-CoV-2 infection in the respiratory and GI tracts. Prevention of further infection may result given 79,found that nasal application of IgA and other antibodies prevented future infections of the respiratory tracts. Notably, IgA falls rapidly after a Covid-19 infection in those under 60 years old, and has yet to be reported in older patients [80].

Let’s consider some the key lingering morbidities in viral infections, and specifically Covid-19, and how a stem cell therapeutic candidate may successfully treat these conditions. This is especially important given the evidence suggests that stem cells in the respiratory tract and elsewhere may be compromised by SARS-CoV-2, and account, at least partially, for increased inflammation and repair deficits in Covid-19 patients [81,82].

## RE stress and protein misfolding- HSP and chaperones

Evidence suggests that ER stress and sustained UPR signaling significantly contribute to the pathogenesis of inflammatory disorders and viral infections and can increase the severity of these indications [83]. Viruses interact with the host UPR to maintain an environment of persistent infection, and ER stress has been suggested as a drug target in Covid-19 [84]. In previous studies, ER stress as measured by stress granule formation was reduced by the application of stem cell released molecules [26,85].

## DNA and protein damage – antioxidants

Viruses have a small genome size, and therefore rely on host machinery in order to perform the essential events facilitating their replication. As the SARS-CoV-2 virus hijacks the host functions, DNA and protein damage can result [86]. Large changes were observed in protein phosphorylation 24 hours after SARS-CoV-2 infection, highlighting the degree to which the virus makes use of the host post-translational regulatory pathways to promote rapid changes in cellular signaling to suit its spread [87]. The secretome from ADSCs is loaded with antioxidants, chaperones, and heat shock proteins to prevent and remediate DNA and protein damage [26].

## Lung fibrosis

Pulmonary fibrosis has been reported in severe Covid-19 patients [88] and can cause long term pulmonary dysfunction and eventual death. Evidence suggests that stem cells in the respiratory tract, including the alveoli, and elsewhere may be compromised by SARS-CoV-2, and account, at least partially, for increased inflammation and repair deficits [89–92,94–95,97]. ADSCs and the molecules they release have been found mimic stem cell function (Maguire, 2019) and to reduce pulmonary fibrosis in a number of animal models [93].

## Blood vessel damage and anti-coagulant properties

Autopsies of Covid-19 patients reveal significant endothelial damage not typical of myocarditis, and possibly not associated with inflammation. Cardiovascular morbidities in 60% of patients surviving severe Covid-19 are characterized by continued myocardial inflammation, lower left ventricular ejection fraction, remodeling of heart tissue, and likely scarring independent of preexisting conditions [96]. ADSC injections have been found to be safe in cardiovascular patients and improve heart function by reducing inflammation and fibrosis [98]. While bone marrow mesenchymal stem cells may stimulate coagulation of the blood [99], the secretome (conditioned media) from adipose mesenchymal stem cells (ADSCs) does not have this effect [100].

## Autoantibody production in infection and vaccination

Both infection and vaccination can produce autoantibodies that are damaging to the patient. [101],found that Covid-19 patients exhibit significant increases in autoantibody reactivities as compared to uninfected individuals in the exoproteome, with a high prevalence of autoantibodies against immunomodulatory proteins, including cytokines, chemokines, complement components, and cell-surface proteins. Likewise, Covid-19 vaccines have been found to elicit damaging autoantibodies, requiring in some cases the need for life-saving, drastic therapeutic intervention, including plasma exchange, corticosteroids, rituximab to destroy a subset of B cells [but, importantly, not gut B cells that quell inflammation in autoimmune disorders; see 102], and Caplacizumab to break the abnormal blood clotting [103]. Autoantibody production can be a long-term event, and lead to numerous physical ills, including neurodegenerative diseases [104]. Mesenchymal stem cells have been shown to limit B cell mediated autoimmunity [105] and reduce autoantibody levels in serum [106], and along with diet [107,108] may be an important therapeutic strategy in limiting autoantibody production and autoimmune diseases, often a part of the Covid-19 sequalae.

## Safety and efficacy considerations: ADSCs preferred Over BMSCs

When addressing safety and efficacy concerns of stem cells, we must consider tissue-specific stem cells [109]. Choosing the appropriate stem cell type to match the condition to be treated is critical not only to efficacy, but most importantly, safety of the therapeutic. Beyond the genetic and epigenetic factors that influence stem cell phenotype as embryonic stem cells differentiate into somatic stem cells [110], the immediate niche of the stem cell will have profound influence on the cell’s phenotype [109]. As described below, I discuss the appropriate use of adipose derived mesenchymal stem cells (ADSCs) for Covid-19 and other viral diseases, compared to bone marrow mesenchymal stem cells (BMScs) that exhibit some potentially dangerous characteristics that should limit their use in therapeutic development.

The complexity of the bone marrow (BM) niche can lead to many stem cell phenotypes, whether we consider hematopoietic stem cells (HSCs) or bone marrow mesenchymal stem cells (BMSCs). Here I will discuss the properties of BMSCs, not HSCs. Because of the complexity, many BMSC phenotypes exist, including disease causing phenotypes that are varied and hard to distinguish [111] – a part of the problem in using BMSC for therapeutic development. This complication, unlike that for ADSCS, includes recirculated cells, particularly recirculated cancer cells. Once a tumor cell disseminates into the BM, the cancer cell often displays phenotypic characteristics of BMSCs rendering cancer cells difficult to distinguish from BMSCs [112]. BM is a site of BMSCs that may differentiate into HSCs [113] and recirculating blood cells that may differentiate into BMSCs [114,115]. BMSCs are also found outside of the niche in peripheral blood [116] and home into sites of injury [117] and cancer tissue where they are educated into becoming a pro-cancerous phenotype [118]. Recirculated melanoma and myelogenous leukemia cells [119] in BM interact with BMSCs to change the phenotype of the BMSC to one that is cancer promoting by enhancing their proliferation, migration, and invasion and altering the production of proteins involved in the regulation of the cell cycle [120]. Indeed, melanoma tumor cells start to disseminate to BM during the initial steps of tumor development [121]. In breast cancer patients, detection of recirculated cancer cells that disseminated in BM predicts recurrence of the cancer [122]. Cancer cells can fuse with BMSCs and change their phenotype [123], or release exosomes to change the phenotype of BMSCs to cancer promoting [124]. Indeed breast tumor cells fuse spontaneously with bone marrow mesenchymal stem cells [125]. This fusion may facilitate the exchange of cellular material from the cancer cell to the BMSC rendering the fused cell more oncogenic [126]. Further, others have found the same result of this fusion and exchange of cellular material, which has been found to increase metastasis. For example, 127,found that human hepatocellular carcinoma cells with a low metastatic potential exhibit a significantly increased metastatic potential following fusion with BMSCs *in vitro* and in xenograft studies. In the end, the BMSCs and their molecules/exosomes, having been conditioned by tumor cells, were found to increase the probability of cancer in human patients [128]. The various phenotypes of BMSCs, including the cancerous phenotypes are difficult to distinguish [36]. In contrast, even ADSCs derived from cancer patients have been found to be safe for therapeutic development [66].

One of many reasons why ADSCs are preferred compared to BMSCs is that ADSCs express a low level of major histocompatibility complex (MHC) class I molecules and do not express MHC class II and costimulatory molecules. Even the exosomes of BMSCs express MHC class II proteins [129]. These problems in BMSCs are amplified when using donor, allogeneic BMSCs that have been replicated many times, essentially aging the cells, during expansion to develop the therapeutic. This is in contradistinction to ADSCs. Critically, when comparing experimental data of BMSCs to ADSCs from the same human donor, “ADSCs have a “younger” phenotype,” according to stem cell scientists [130]. Indeed, Burrow et al found that BMSCs have, among other negative attributes compared to ADSCs, an increased level of senescence compared to matched ADSCs. Senescent cells develop the senescence-associated secretory phenotype (SASP), a pro-inflammatory set of molecules where the local tissue effects of a SASP or specific SASP components have been found to be involved in a wide variety of age-related pathologies *in vivo* such *as* hyperplastic diseases, including cancer [131]. Whereas the use of BMSC transplants has a history of medical adverse events, including the induction of cancer in the recipient (Maguire, 2019), fat grafting, along with its constituent ADSCs, have a long history of safety in medical procedures dating back to 1893 when the German surgeon Gustav Neuber transplanted adipose tissue from the arm to the orbit of the eye in an autologous procedure to fill the depressed space resulting from a postinfectious scar [132]. Fat grafting’s long history of being safe, regardless of the harvesting techniques used in patients [120,133], has been recently reviewed by physician-scientists at Baylor College of Medicine [134]. Furthermore, physician-scientists at Stanford University School of Medicine have recently reviewed the safety and efficacy of using ADSCs to augment the outcomes of autologous fat transfers [135]. 136,have found that ADSCs and fat grafting for treating breast cancer-related lymphedema is safe and efficacious during a one year follow-on, where patient-reported outcomes improved significantly with time. In a randomized, comparator-controlled, single-blind, parallel-group, multicenter study in which patients with diabetic foot ulcers were recruited consecutively from four centers, ADSCs in a hydrogel was compared to hydrogel control. Complete wound closure was achieved for 73% in the treatment group and 47% in the control group at week 8. Complete wound closure was achieved for 82% in the treatment group and 53% in the control group at week 12. The Kaplan–Meier (a non-parametric statistic used for small samples or for data without a normal distribution) median times to complete closure were 28.5 and 63.0 days for the treatment group and the control group, respectively [137]. Treatment of patients undergoing radiotherapy with adult ADSCs from lipoaspirate were followed for 31 months and patients with “otherwise untreatable patients exhibiting initial irreversible functional damage” were found to have systematic improvement or remission of symptoms in all of those evaluated [138]. In animal models with a full thickness skin wound, administration of ADSCs, either intravenously, intramuscularly, or topically, accelerates wound healing, with more rapid reepithelialization and increased granulation tissue formation [139], and topically applied the ADSCs improved skin wound healing by reducing inflammation through the induction of macrophage polarization from a pro-inflammatory (M1) to a pro-repair (M2) phenotype [140].

## Therapeutic and preventative regimens using ADSCs

The use of our proprietary blend of the secretome from ADSCs and their differentiated cell types, fibroblasts, is supported by safety studies [27] and efficacy data [26]. Total secretome, not just the extracellular vesicle (EV) fraction, is used because soluble proteins and the EV fractions have been characterized by 384 of 781 proteins being mutually exclusive, and total secretome is more effective than just the EV fraction [141]. The superiority of using ADSCs versus other stem cells types has been described [Maguire, 2019; 129, 142]. Fibroblast secretome provides many important benefits to tissue repair (Maguire, 2019) and also possess antimicrobial properties, including antiviral replication actions [143,144]. The intravenous administration of ADSCs has been successfully used for ARDS in Covid-19 [145], but when BMSCs have been used in Covid-19, compliment dysregulation causes severe clotting, IV cell administration may result in aggregating or clumping in the injured microcirculation and further carries the risk of senescence, mutagenicity, and oncogenicity, and possible cellular aging effects in the immune system [31], which do not exist by treating with nebulized ADSCs secretome. Another advantage of ADSC exosomes over MSCs is the possibility of storing them for several weeks/months allowing their safe transportation and delayed therapeutic use. Preclinical testing can use, for example, a mouse model of Covid-19, where a genetically modified SARS-CoV-2 virus is constructed to bind the mouse ACE2 receptors to infect the cells [146]; i.e. a recombinant virus (SARS-CoV-2 MA) that could utilize mACE2 for entry. The mouse-adapted SARS-CoV-2 model demonstrates age-related disease pathogenesis similar to that found in human COVID-19 infections.

The therapeutic strategy may provide an unusually low risk and high reward ratio – because human identical molecules are used to normalize immune function and physiology. Nasal delivery through nebulization is used to reach respiratory tracts, including alveoli. Tissue remodeling of the bronchi has been associated to a reduction in secretory IgA (sIgA) levels at the mucosal surfaces in human patients. In addition, sIgA from submucosal glands are snared into mucus plugs, limiting the natural immune response [147]. Suboptimal antibody responses, including in the aged, may facilitate SARS-CoV-2 infection [148]. ADSC secretome facilitates an IgA response to an antigen in the aged by reducing immunosenscence [78], and reduces lung inflammation and promotes tissue repair through education of lung macrophages to the M2 phenotype [149].

Two-phased therapeutic strategy – commercialized technology exists to rapidly determine viral titer in patients at the point-of-care setting [150] so that once the patient has cleared the virus, the second phase therapeutic can then be administered to resolve the inflammation and begin tissue repair. Thus a nasally self-delivered therapeutic, obviating the need for bringing the patient into a clinical setting, may be used to prevent infection, and in the clinical setting, once the infection has cleared, may be used to mitigate Covid-19 related morbidities and mortality.

## Systems therapeutic approach

When the term “magic bullet” is searched in PubMed, 886 results are found that discuss profound, specific results for a given medical intervention. The “magic bullet” thinking is reductionist. What I’m describing here instead is a “systems therapeutic” for “physiological renormalization” (26) so that the body can better fight the viral infection and more quickly and efficiently resolve the ensuing inflammation that induces much of the damage to the body. The therapeutic would consist of multiple molecules, targeting multiple pathways, often at multiple levels of the body. An example is the result for combining fiber in the diet, acting at the level of the gut to facilitate the function of bacterial symbionts that enhances T cell function [69], along with “checkpoint inhibitors,” acting at T-cells, that has been found to enhance the cancer cell destroying capabilities of a checkpoint inhibitor drug used to treat melanoma [151]. Thus, the strategy is to use a combination of molecules as a systems therapeutic [152–154] that are derived from: 1. The molecules naturally produced by some of our stem cells that act as a part of and regulate the immune system, and 2. Feeding the body a diet biased toward plants with soluble and insoluble fiber that will positively renormalize the immune system (27), both of which will in effect renormalize the immune system to better fight the SARS-CoV-2 virus and quell the ensuing, damaging inflammation.

So what can we do now why scientists are working on new therapeutic for infection by SARS-CoV-2, including stem cell based antimicrobial therapeutics to fight coronavirus Covid-19? Thinking in terms of the systems therapeutic concept, consideration of one’s exposome is critical to better preventing and mitigating the effects of Covid-19 or other infections (Maguire, 2020). In the context of stem cell function alone, diet affects stem cell physiology such that, for example, sustained mTOR signaling may account for some of the decline in stem cell function with age [155]. mTOR can be elevated by high-protein, animal based diets [10,156]. First, follow public health measures as instructed by your local health authorities. Second, eat well, including a predominantly whole food plant based diet that provides a rich variety of healthy molecules, including soluble and insoluble fiber that will set the immune system in a state to better fight the infection and resolve the inflammation [69] and may induce mechanical autophagy in the gut to help clear infection [157]. And, like ADSCs that release a rich variety of antioxidant types (63), eating predominantly plants will also provide a rich variety of antioxidants to setup the antioxidant cascade [158] to help quell the viral infection [58,155,159,160]. The plant based diet will also be a good source of linoleic acid [161,162], that can induce T-cell function and potentially better clear the infection, and reduce inflammation [163]. Being careful to control diet as part of one’s exposome, all of the molecules we’re exposed to in life, can increase lung function and immunity [164], and likely lead to better outcomes in preventing and mitigating the effects of Covid-19 (27). Because diet affects stem cells function [155], these same diet strategies may also be important for enabling better endogenous stem cell function important in preventing and mitigating Covid-19. Thus, as we’ve seen in cancer treatment where checkpoint inhibitors are used to induce “physiological renormalization” of the immune system, and fiber is dosed to the patient too to provide a “systems therapeutic” enhancement of the checkpoint inhibitor, in viral infections too we can induce “physiological renormalization” with therapeutics derived from stem cells and their molecules, along with a diet that is plant based biased to enhance the immune system and endogenous stem cell function that induces a “systems therapeutic” approach to infection. Thus, using a systems therapeutic approach, including stem cell released molecules as a therapeutic, along with plant-biased diet to renormalize physiology may better prevent and mitigate the effects of Covid-19.
